# Merkel Cell Carcinoma of the Head and Neck: Challenges in Diagnosis and Therapy

**DOI:** 10.1155/2013/427984

**Published:** 2013-09-10

**Authors:** Brett A. Miles

**Affiliations:** ^1^Otolaryngology Head and Neck Surgery, Icahn School of Medicine at Mount Sinai, New York, NY 10029, USA; ^2^Oral and Maxillofacial Surgery, Icahn School of Medicine at Mount Sinai, New York, NY 10029, USA

Since the original description of the trabecular carcinoma of the skin, now known as Merkel cell carcinoma (MCC), by Toker in 1972, this malignancy has provided significant challenge to clinicians. With an increasing incidence and a known poor prognosis for advanced lesions, the disease continues to challenge physicians in the present time. New evidence indicates that not only the incidence is increasing in the overall population but also certain groups of patients such as those taking medications or immunosuppressed are at increased risk. Additionally, patients affected by MCC may be at risk for other malignant diseases. Therefore, it is likely that MCC will continue to challenge clinicians in the future.

Current clinical questions regarding the optimal management of MCC include surgical challenges such as histologic diagnosis, sentinel lymph node biopsy, and margin control, as well as issues related to reconstruction. Radiation oncologists continue to evaluate the appropriate strategy for this rare disease in terms of locoregional control and appropriate dosing strategies. Medical oncologists continue their quest for effective treatments for this rare disease, which does not lend itself to randomized controlled trials due to the small number of eligible subjects.

Recent new data regarding the analysis of MCC has resulted in a dramatic increase in our knowledge regarding this disease. The discovery of the Merkel cell polyomavirus by Feng, et al. and the subsequent work of Touze et al. which showed high antibody titers against the virus (anti-MCPyV) correlating to improved survival have provided us with new data with which to target this disease. Work in viral detection and protein expression continues to further elucidate the role of the virus in the genesis of MCC. The development of viral vaccines and targeted agents for the treatment of MCC is ongoing. 

The purpose of this special issue is to update the clinician on the current literature regarding Merkel cell carcinoma and provide a consolidated review of this disease for the clinician. A historical perspective as well as the current management strategies will be reviewed. Recent data will be presented regarding current diagnostic methods, and pathological evaluation as well as an evaluation of surgical, radiotherapeutic, and chemotherapeutic strategies employed in the treatment of MCC will be examined in the articles within the issue. In addition, future areas of research will be identified to allow the reader to plot the trajectory of our current understanding of this disease and where scientific and clinical research efforts are likely to yield advances in the future.

*Brett A. Miles* *Brett A. Miles*

